# Innovating the practice of epilepsy care to improve patient outcomes: a model for community health worker integration

**DOI:** 10.3389/fneur.2025.1654687

**Published:** 2025-10-07

**Authors:** Elaine T. Kiriakopoulos, Felicia Chu, Barbara C. Jobst, Rosemarie Kobau

**Affiliations:** ^1^Department of Neurology, Geisel School of Medicine, Dartmouth College, Hanover, NH, United States; ^2^The Dartmouth Institute for Health Policy and Clinical Practice, Hanover, NH, United States; ^3^The HOBSCOTCH Institute, Dartmouth Health, Lebanon, NH, United States; ^4^Department of Neurology, University of Massachusetts Chan Medical School, Worcester, MA, United States

**Keywords:** epilepsy specialist teams, patient centered care, community health workers, health equity, disparities, social determinants of health, integrated care teams

## Abstract

There is substantial evidence that community health worker (CHW) interventions can lead to improved health outcomes. However, their integration into specialty care teams—particularly within epilepsy care—is still limited. Embedding CHWs onto epilepsy center teams presents a promising opportunity to address health inequities by incorporating whole-person patient centered care and addressing social determinants of health (SDOH). The conceptual model introduced in this manuscript highlights the importance of collaboration between epilepsy centers, medical centers, health systems, community partners and nontraditional CHW professional workforce to drive systemic change. We share a process model that supports the pragmatic integration of CHWs onto epilepsy care teams and the implementation of standardized SDOH screening taking into account existing multidisciplinary professional roles and common epilepsy center frameworks for delivering care.

## 1 Introduction

Epilepsy impacts 3.4 million people in the United States (1). There are individual, community and health-system barriers to optimal epilepsy management, and consequences of epilepsy are further exacerbated by the substantial geographic, socioeconomic, racial and ethnic disparities which exist in neurologic specialty care (2–5). Community Health Workers (CHWs) function as liaisons between healthcare systems, social services, and the community to facilitate healthcare access and care delivery (6, 7). Substantial research suggests CHWs can effectively assist patients with some conditions (e.g., asthma, diabetes, cancer) with care coordination, disease self-management, improving health-enhancing behaviors, and reducing barriers to accessing care (6, 7). There is a burgeoning interest in the integration of CHWs into medical center care settings to amplify coordinated care and manifest stronger clinical-community linkages, to facilitate improvements in the quality of patient care and to reduce health disparities (6, 8). Knowledge around disparities in epilepsy care and outcomes suggests that to address gaps in care, focused effort be placed on solutions at the point of patient care (9–13). Yet, scant research has explored the integration of CHWs in neurologic clinical care, including in epilepsy center care. In response to stakeholder recommendations regarding the potential benefits of using certified health educators in epilepsy care, the US Centers for Disease Control and Prevention Epilepsy Program developed a community health worker curriculum for epilepsy self-management (2) and later supported formative and effectiveness research in this area (14, 15).

Growing research (6–13) on the role of CHWs in managing chronic conditions suggests that CHWs positioned in an epilepsy center could support people with epilepsy with their needs around improved care coordination, health education, empowering patients to manage their disease including delivering standardized epilepsy self-management programs, and with addressing unmet social needs that impede health. Epilepsy center clinicians have identified assisting patients with transportation and medication needs as a priority role for CHWs (14, 15) and shared that it would be helpful to have a CHW on an epilepsy center team serve as a communication liaison between the provider and patient during and beyond clinic appointments to follow up on medication access, transportation to the next clinic visit, or medical testing. Despite the possibility of overlap between social work and CHW roles in some domains of non-medical care, prior epilepsy center clinician perceptions endorse the possibility for the roles to work in synergy (14, 15). Importantly, prior efforts have demonstrated acknowledgement of pervasive care gap needs requiring action and the benefits of integrating a CHW into the epilepsy team align between physicians, nurses and social workers (14, 15).

**Figure 1:**
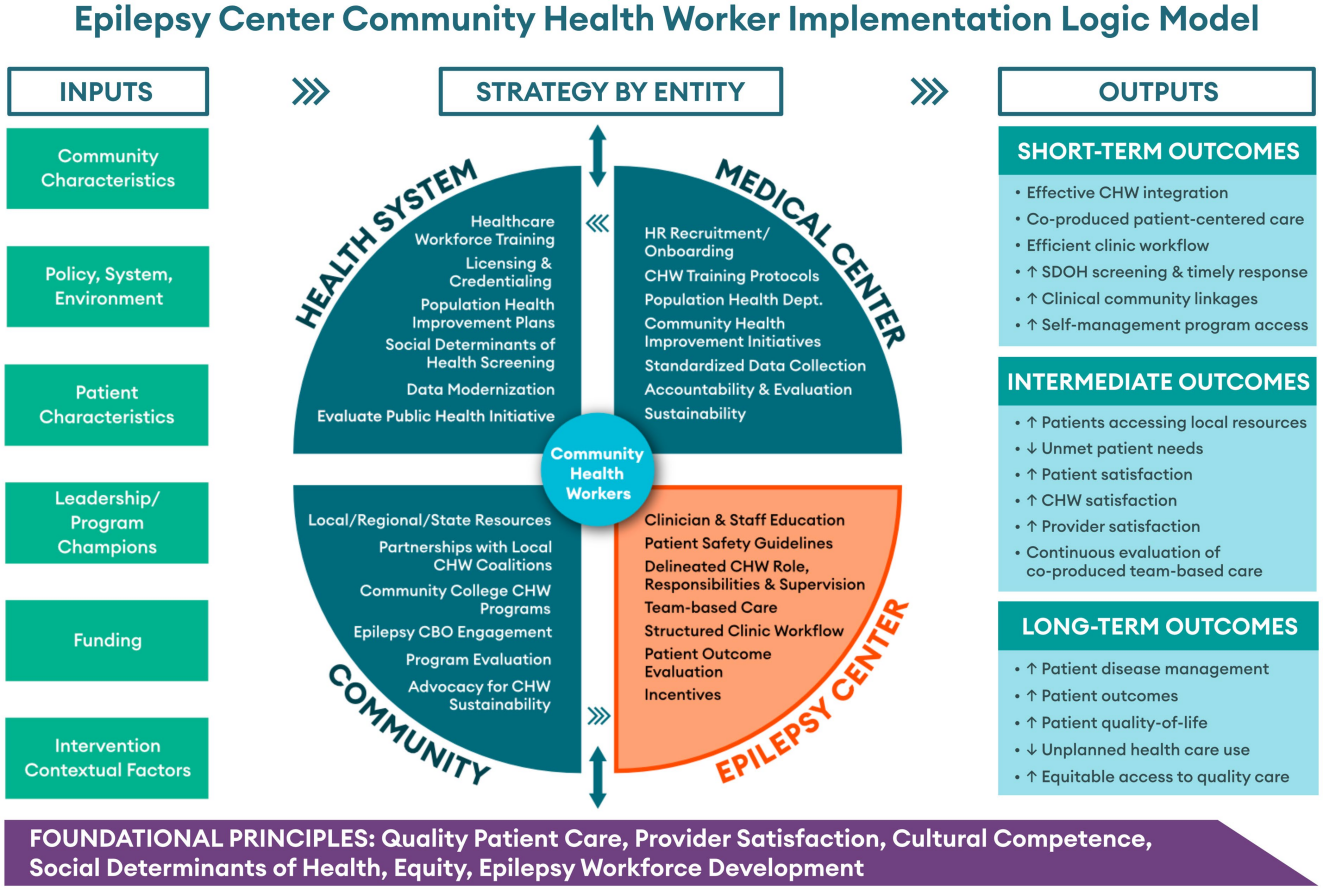
Logic model describing integration of a CHW within an epilepsy center clinical team, accounting for inputs, strategies, contextual factors, and expected outcomes.

**Figure 2:**
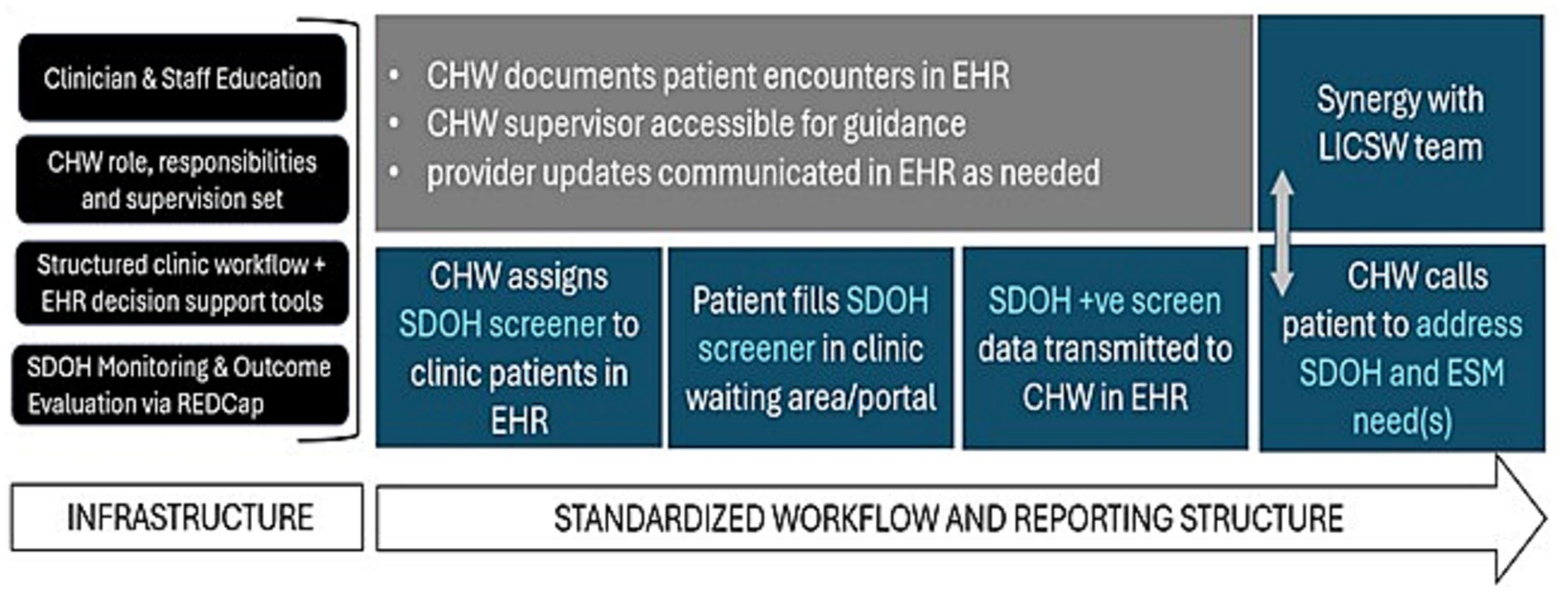
Process model for epilepsy center CHW integration.

This paper extends formative studies (14, 15) of clinician perceptions and readiness for the integration of CHWs onto epilepsy center care teams. The objective of this manuscript is to provide epilepsy centers with a logic model and process model (i.e., “guide”) for integrating a CHW as a care team member and implementing a clinic-based intervention aimed at decreasing disparities and improve health outcomes for people with epilepsy. The logic model we present (Figure 1) considers the complexities and interplay of inputs (e.g., healthcare system champions; funding), and strategies (e.g., education, recruitment) that can serve as enabling factors to effective CHW integration. The process model presented (Figure 2) highlights key infrastructure and workflow components, subject to adaptation by different epilepsy centers relative to their existing capacity.

## 2 Logic model for CHW integration

Our logic model was developed with consideration of critical components (i.e., foundational principles and processes) related to the integration of a CHW within an epilepsy center clinical team, accounting for inputs, strategies, community contextual factors, and expected outcomes. Underlying the model are the foundational principles of patient care, provider satisfaction, cultural competence and epilepsy workforce development; also considered are social determinants of health and equity (Figure 1). The logic model (Figure 1) flows from left to right and illustrates the directionality of system components and relationship between inputs and outputs.

As members of multidisciplinary epilepsy teams, CHWs have potential to increase epilepsy center capacity by assuming non-clinical responsibilities that other healthcare professionals can only partially address, if at all, due to priority clinical duties. Fundamental to successful CHW integration are inputs including community need, organizational champions, and identification of potential funding streams. Key strategies to successful integration of a CHW into an epilepsy center team involves education of clinicians and staff on the roles and responsibilities of a CHW, supervision of the CHW, and a clear plan for structured clinic workflow and the implementation of team-based care. It is paramount to cultivate the idea of CHWs as members of health care teams. Evaluation of outcomes and incentives to propel the team-based approach, including feedback from patients working with a CHW, should be shared regularly to facilitate an informed and invested epilepsy center team. Further, at institutions where CHW programs exist in comparable medical specialty clinics, there are opportunities for sharing CHW workflow tools/guidelines, and proven sustainability mechanisms, within an institution, ensuring the administrative supports are informed and engaged in bringing CHW support to epilepsy center teams.

## 3 Process model for CHW integration

Our process model focuses on infrastructure, workflow and oversight—inclusive of epilepsy center, medical center, and health system components (Figure 2)—embedded within the broader community context that can enable action. Core components of this model include clinician and staff education, ensuring a well-defined CHW role, structured clinic workflow, standardized SDOH screening and monitoring of outcomes. Incorporation of embedded EHR decision support and reporting tools can optimize communication between team members, however screening can be done independent of the EHR so long as communication among epilepsy center team members is consistent (for example, regular report out on CHW efforts at team rounds), timely, and contiguous with patient care. In the case of an epilepsy center not having an integrated social worker in place, options for synergistic patient support could include the CHW working together with advanced practice providers (APRN, PA) to ensure patient needs met. Representative case examples are presented in Table 1.

**Table 1:** Representative case examples of integrated CHW intervention and resource supports shared to meet patient SDOH needs in the epilepsy center clinic.

Example case	Workflow for identification of SDOH need	CHW action and resources provided	Outcome and follow up
RS, 47 year old Veteran with uncontrolled epilepsy	CHW assigned SDOH screener in EHR patient portal 2 weeks prior to clinic visit. Patient completed screener from home prior to clinic visit and indicated need for assistance with transportation.	Telephone outreach to patient by CHW to provide a New Hampshire (NH) state resource, Home Healthcare, Hospice & Community Services; www.hcsservices.org/services/transportation/ which is available to provide people with transportation needs to medical appointments at Dartmouth Hitchcock Medical Center and the state VA Medical Center.	CHW follow up call to ensure patient travel support to his clinic visit was in place. CHW documented transportation need met in EHR. CHW reviewed EHR to see patient did attend scheduled clinic appointment.
MN, 68 year old woman with uncontrolled epilepsy who had recently relocated to rural NH	CHW assigned SDOH screener in EHR patient portal. Patient did not fill out screener prior to appointment, and thus patient was provided with a tablet by CHW at clinic check in to complete the screener while she was in the clinic waiting room. Patient filled out the screener while waiting to see her clinician and indicated food insecurity and feeling isolated secondary to not driving and being new to the area.	CHW able to connect with patient in person following her clinic visit to provide resources to assist her with identified needs. Resources provided to patient: *Mobility Advanced Transit* which provides door to door transportation in the NH region, inclusive of travel to medical appointments, as well as for personal errands (grocery store, shopping center, pharmacy, library, etc.) Connection to NH Upper Valley Senior Center which could provide *Meals on Wheels* services to the patient. *Supplemental Nutrition Assistance Program (SNAP)* benefit resources were shared. *NH Upper Valley Community Food Market* resource was provided (https://uppervalleyhaven.org/programs/food-market/) Resource for a *local library social group* for people who enjoyed knitting was shared to assist with social connection to reduce isolation as patient expressed an interest in knitting and crafts. Resource connection to the monthly online accessible *medical center support group meeting for people with epilepsy* was provided.	CHW follow up to ensure patient was able to connect with resources provided for transportation, and able to secure needed food supports available through the provided resources. CHW documented patient needs and resource response in EHR for provider and LICSW awareness. CHW continued to make a weekly call to the patient to help with lessening isolation and to support the patient’s efforts to grow her social connections in her new community.
PW, 28 year old man with uncontrolled epilepsy and cognitive dysfunction limiting his ability for employment	CHW assigned SDOH screener in EHR patient portal and provided opportunity for it to be filled out on a tablet in clinic waiting area. Patient not able to complete. Provider connected the patient with the CHW in-person following the patient’s clinic appointment to assist with needs for completion of disability paperwork and to share the patient’s cognitive challenges that could benefit from self-management program support.	CHW met with patient following the appointment to help him with access to online disability form information and with filling out the required application. CHW informed provider and LICSW of the steps taken to assist with disability application via EHR communication. CHW provided the patient with her contact information and scheduled a follow up call with the patient.	CHW reached out to follow up with patient regarding disability application outcome. CHW extended further support to the patient by providing information and connecting the patient to an evidence based epilepsy self-management program (HOBSCOTCH, HOme Based Self-Management and COgnitive Training CHanges Lives) that could help him to address managing his disease and the comorbid cognitive symptoms.

### 3.1 Recruitment and hiring

Recruitment of CHWs can be multifaceted (16). At individual epilepsy centers, individuals already working as a patient care assistant, licensed practical nurse, or certified nursing assistant hold potential to train to fill a CHW role. Ample opportunities exist to identify licensed CHW candidates by partnering with epilepsy-serving community partners (17) and local, state, and national CHW coalitions and professional organizations. A variety of methods including the involvement of medical center human resource partners as recruiters, internal medical center job posts, flyers at community college job fairs, email list serves connected to healthcare job seekers, social media posts and notifying the epilepsy patient population about the opportunity to train in a CHW role can all aid with recruitment.

Integrating CHWs at epilepsy centers will require clear, well-defined guidelines for selecting suitable candidates for addition onto a multidisciplinary team. Structured job interviews that incorporate case scenarios can help to assess key personality traits, such as listening skills, empathy, and a nonjudgmental attitude, offering valuable insights into a candidate’s potential future performance. CHWs should be able to communicate effectively with clinicians (physicians, nurses, APP, LICSW) via telephone or electronic medical record charting and collaborate in person during multidisciplinary rounds.

### 3.2 Training

Training programs for CHWs vary in scope and implementation of core training standards and accreditation by state (18, 19). The National Association of Community Health Workers provides resources for CHW networks and associations across 38 states and their related state training and certification programs.^1^ Epilepsy-specific trainings are meant to be delivered to individuals as a complement to core CHW training programs, and are best suited to CHWs who have experience in the profession and/or accreditation in their home states. Our experience has revealed that CHW training development for clinical settings such as an epilepsy center need to be built on strong academic clinical partnerships that involve input from both experienced clinical CHWs and other partners. We garnered input from our medical center leaders that support the institution’s population health goals and CHWs working throughout the Dartmouth Health system to aid in the development of our training program for CHWs whose work would focus on the care of patients in a specialty clinic.

Training curricula for CHWs specific to epilepsy is available through two principal mechanisms. The first is the ability to share the CDC Community Health Worker’s Training Curriculum for Epilepsy Self-Management developed for broad dissemination in 2017. This training manual is accessible for use by various instructors, health educators, organizations and agencies.^2^ The second is via a four-hour accredited online “Overview of Epilepsy & Self-Management” interactive live training developed at Dartmouth Health’s Community Epilepsy & Self-Management Training Center.^3^ This training facilitates learning in small group training sessions tailored to individual epilepsy centers who are integrating a CHW onto their multidisciplinary team, or community organizations who wish to train CHWs to serve in diverse epilepsy settings, or through accessible Area Health Education Center programming. Trainings are taught by multidisciplinary faculty with subject matter expertise including epilepsy specialist physicians, psychologists, masters of public health educators, nurses and CHWs who have completed epilepsy specific accredited training and have experience in a clinical setting. Core training objectives related to living with and managing epilepsy include understanding the diagnosis of epilepsy, different types of seizures and how they impact people; available treatments for epilepsy and seizure first aid; increasing knowledge of the challenges and comorbidities associated with living with epilepsy (mental health, cognitive dysfunction, stigma, employment, transportation); increasing knowledge surrounding self-management domains and opportunities for connecting people to evidence-based epilepsy self-management support; growing knowledge on resources for people with epilepsy and how to empower patients to make use of these resources. Supplemental epilepsy-specific courses for CHWs on Social Determinants of Health, Psychosocial Health and Stigma, and Barriers to Medication Adherence, delivered in three 90-min live online interactive training sessions are also available to augment epilepsy specific knowledge for CHWs serving at epilepsy centers. Post-training support for CHWs and the centers integrating CHWs is provided through Dartmouth Health’s Community Epilepsy & Self-Management Training Center team.

### 3.3 Roles and responsibilities

CHWs need a clearly defined role that is well understood and respected by the existing epilepsy clinical team, and they need on-going functional supports from the health system and from the epilepsy center teams they become embedded within. Critical to the success of CHW role integration is fostering a sense of identity as an active contributor in the care of patients, with specific roles for patient support delineated, alongside those of epilepsy center clinicians and support staff (14, 15).

A standardized process to proactively identify patients who would benefit from CHW support is important for growing the CHW patient panel across multiple epilepsy providers. Feedback from epilepsy center stakeholders, including administrative and clinical leadership as well as frontline providers, will allow for the identification of service needs a designated CHW will assume as responsibilities, and allow for defining ideal patients (for example, presence of comorbidities, complex treatment regimens, increased disability secondary to disease) for referral to CHW services.

### 3.4 Supervision and support

Identifying a primary supervisor may differ amongst epilepsy centers, depending on the availability and suitability of existing team members and CHW infrastructure which may already exist at a given medical center. Irrespective of the individual placed in the supervisory role, the components of a solid infrastructure that includes structured clinic workflow, electronic health record decision support tools, standardized monitoring and evaluation of outcomes will be necessary (Figure 2) in order to integrate a CHW effectively onto an epilepsy center team.

Secondary to the integration of CHWs onto primary care and other specialty teams over the past two decades, medical centers may have infrastructure in place that can be utilized as a resource for electronic health record decision support, monitoring of outcomes and partnered supervisory support of an epilepsy center CHW. In the absence of existing medical center support, there are context-dependent supervisory roles that can enhance workflow amongst the epilepsy team. Within most epilepsy centers, a clinical social worker or epilepsy nurse or physician’s assistant advanced practice provider are likely to be well-positioned to assist with CHW supervision. The supervisory approach for a CHW requires tailoring to their specific needs, scheduled one-to-one discussion of caseload, and providing security for an embedded CHW to have access to joint problem solving surrounding individual patient needs. Supervisors should be prepared to function as CHW Champions, given the relative novelty of an integrated CHW role on epilepsy center teams. The supervisor will be key to providing ongoing communication and insights to epilepsy center clinicians and staff regarding the value and contributions of the integrated CHW role, and to ensure any individual resistance about the CHW role is addressed.

### 3.5 Professional development

As in other health care professional roles, developing pathways for professional development of CHWs as they are integrated on epilepsy center teams is vital to retaining a knowledgeable trained team member, and brings potential for expanding CHW presence at an epilepsy center. Advancement factors may consist of additional training, certifications, taking on a role in mentoring new CHWs, participating in the delivery of training sessions, or more formal education (20, 21). One example of a well-suited professional development opportunity is for a CHW serving at an epilepsy center to be provided with the opportunity to train to become certified to deliver evidence-based epilepsy self-management (ESM) from the CDC’s Managing Epilepsy Well catalog of programs. The core epilepsy CHW training described above introduces CHWs to the ESM programs so they are equipped to share access to programs from their CHW positions. Providing the opportunity to train and become certified to deliver these programs expands the CHW professional role at an epilepsy center to include standardized education delivered through ESM programming and can not only enhance their contribution to the epilepsy center team, but more importantly bring a new level of support to the patients they support. Further examples include integrating the CHW in Seizure First Aid education and contributing to epilepsy education conferences that engage patient communities and community social service providers.

### 3.6 Relationships with communities and health systems

Epilepsy centers, epilepsy and CHW professional organizations (e.g., American Epilepsy Society, the National Association of Community Health Workers, regional CHW coalitions) and community partners (e.g., Epilepsy Alliance, Epilepsy Foundation, Epilepsy Association) must work synergistically to assess and improve organizational and health systems readiness for sustainable integration of CHWs into epilepsy care streams. Implementing and adapting early integration models will lead to improving the adaptability and preparedness of epilepsy center teams to integrate CHWs into coordinated care delivery models.

A key challenge of developing large-scale CHW programs targeting a specific chronic disease, epilepsy, is that stakeholders in leadership positions (National Association of Epilepsy Centers, American Epilepsy Society) need to establish and maintain constructive relationships with the policy makers and payors steering national health systems, as well as with individuals embedded in the community. They need to navigate these dynamic relationships over time to build a cohesive effort to allow for the potential population health objectives related to CHW initiatives (i.e., identifying and addressing SDOH, increasing capacity for the delivery of evidence based self-management programming, care access for vulnerable populations) to establish a base of support from which to grow.

### 3.7 Program sustainability

Healthcare financing is increasingly being tied to service value and outcomes as opposed to payment provided in exchange for services rendered. This shift in healthcare financing brings increased opportunities for epilepsy centers to partner in supporting sustainable high-quality CHW services that have proven effective in lowering avoidable costs and increasing patient satisfaction.

Despite the important patient centered work they perform CHWs have long been subject to debate about their value and remuneration. However, as evidence has grown that supports their effectiveness, an emerging consensus exists that in order to achieve sustainability CHWs should be paid fairly for the important service they provide (22). In other chronic conditions, CHWs have mainly been funded under short term non-profit, state or federal grant programs designed for pilot projects and infrastructure development. However, multiple states (e.g., Georgia, Kentucky, Connecticut), reimburse CHW services through Medicaid plans for certain groups.^4^ Previous work by Kangovi et al. (23), described a CHW model that achieved a favorable return on investment for Medicaid payers by effectively responding to SDOH needs; the CHW intervention returned $2.47 for every dollar invested.

Efforts to tap into Medicaid reimbursement of CHW services in epilepsy will require evidence supporting CHW contributions to improved health outcomes as well as appropriate containment of healthcare utilization costs. Epilepsy center champions, CHWs and key partners must work to educate providers, health payers and policymakers around exploring pilot studies to assess the potential benefits that CHWs bring to multidisciplinary teams and the patients they care for.

Next, steps to partner collectively with epilepsy serving professional organizations and state based professional CHW organizations and their licensing bodies to advocate with payors for a structured approach to integrating epilepsy training for CHWs that could lead to improving patient access to CHW services covered by medical insurance. Studies of CHWs engaged by payers in chronic care management have shown evidence of effectiveness in reducing hospitalizations and emergency care visits and containing health care costs (24). Examining healthcare reform-related alternative payment structures and internal funding by provider groups and institutions in anticipation of reduced costs (22, 25, 26) and a return on CHW integration advancement are two potential avenues that must be explored for overcoming sustainable funding hurdles.

Epilepsy clinicians championing CHW value and health system leaders looking to improve outcomes and reduce waste in health care utilization will play a key role in leveraging pathways that allow for CHW programs to take root at their institutions, secure sustainability, and scale to impact health systems. They can do so by focusing on promoting the unique abilities of CHWs as non-medical team members to assist patients in not only resource access but also in understanding the critical need to accept medical advice (for example, employing strategies to aid medication adherence, follow through on testing and clinic appointments, improving self-management behaviors) and monitor their progress, which can lead to a reduction costly hospitalizations, or their ability to complete administrative tasks at a lower cost than when performed by other clinical team members (27).

### 3.8 Example of CHW integration

There are limited examples of CHW integration onto epilepsy center clinical care teams, either independently at an academic medical center, in community clinic settings or through collaboration with local epilepsy social service organizations. One example of successful CHW integration at the University of Texas allowed for CHWs to successfully be integrated into clinical settings in coordination with local Texas Epilepsy Foundation offices. CHWs are trained to use the MINDSET (28–32) decision support program to assess PWE clinical, behavioral, and social metrics and to provide a printed Action Plan of patient-selected behavioral goals, priority social determinants, and recommendations for engagement in evidence-based epilepsy self-management programs. Systematic onboarding and training, inclusive of Dartmouth’s *“Overview of Epilepsy & Self-Management”* training improved CHW knowledge about epilepsy, self-efficacy to provide ESM education to patients, and perceived negative stereotypes about PWE (33). This example lends support for our model with its focus on CHW role delineation, training, and an emphasis on CHWs addressing SDOH and self-management needs in PWE.

## 4 Discussion

Recommendations to train and embed non-traditional providers into epilepsy clinics to address gaps in epilepsy care have been documented for more than a decade (2). Focusing on embedding CHWs into epilepsy care teams in particular, offers a promising way to improve patient outcomes and address inequities. Our logic model highlights broad environmental-, community-, and organizational-level factors for epilepsy centers to consider when planning for CHW integration. Our prior work investigating epilepsy clinician perceptions and readiness to incorporate CHWs on care teams supports the model presented here (14, 15).

Our experience at the Dartmouth Epilepsy Center demonstrates that the model presented here (Figure 2) for integration of CHWs into epilepsy care can lead to effective and efficient care improvements (34). The full integration of a trained CHW in a neurology clinic setting has provided opportunity to systematically screen patients for SDoH utilizing the EMR patient portal to facilitate screener dissemination. Early data demonstrated that epilepsy patients screened positively for SDoH needs three times more frequently when compared to a cohort of patients seen in the general neurology or other subspecialty clinics. In year two of clinic implementation, as part of professional development and to best meet the needs of patients seen in the epilepsy clinic the CHW was trained and certified to deliver epilepsy self-management to patients whose clinical providers felt may benefit from the intervention. The ability for a CHW to address needs has been effective and efficient and the CHW workforce at our center has expanded with 3 Dartmouth clinic based CHWs engaged in SDOH screening and delivering standardized epilepsy self-management (35).

Our process model highlights the need for early and regular epilepsy clinician education and engagement. This reinforces data recently shared around the limited knowledge level which exists across provider groups around CHWs as clinical center team members (14, 15). Notably, the American Epilepsy Society/International League Against Epilepsy-North America Joint Task Force for Epilepsy Health Care Disparities in the United States recently published recommendations for action to address SDOH, knowledge gaps, economic challenges, workforce limitations, and health system deficiencies associated with adverse health outcomes for PWE (25). These recent recommendations also endorse a holistic approach to patient management, inclusive of nontraditional workforce expansion, to both improve patient outcomes and enhance care delivery, and optimize the team’s ability to address barriers (36). CHWs have potential to improve clinical workflow, facilitate access to community supports, and bridge bidirectional communications from clinicians to patients—to potentially close gaps in epilepsy care. These implementation models can serve as a guide for additional formative and effectiveness research by epilepsy partners.

We acknowledge that the potential for pitfalls exists with the implementation of new models of care (37), including in our proposed process model; for example, the presumption of education leading to universal clinician buy in and clinician comfort level with the concept of introducing a non-medical provider onto an epilepsy center team; or the necessary bandwidth available for CHW supervision on teams where social work or advanced practice providers are already stretched thin by their respective workloads in the current healthcare environment. However, despite the potential for hurdles which may arise in trying to expand the composition of an epilepsy care team to include CHWs, the model we share provides a reasonable and practical base for consideration which epilepsy centers can iteratively refine to meet their own contextual needs; including scaling education on the CHW role and ensuring regular reporting on CHW intervention outcomes to clinical teams to grow support from skeptical clinicians who are not accustomed to a non-medical provider on the care team. Our recent efforts in the Northeastern U.S. examining clinician and patient readiness at epilepsy centers for CHW integration (14, 15) support multidisciplinary members of epilepsy center teams are open to considering integration of the skills and services a CHW can provide to people with epilepsy. CHWs can be trained and positioned to collect, analyze, and interpret information on social drivers that influence a patient’s health. Further, by allocating CHWs expertise to identify and address social needs, clinicians are afforded the ability to focus their time and energy on responding to the complex medical issues which often accompany a diagnosis of epilepsy.

### 4.1 Limitations

Factors affecting the applicability of the models described are likely to be varied based on clinical settings despite commonalities which exist at most epilepsy centers. Variations in care delivery by clinician, site and situation require additional characterization in future studies. The effectiveness of the models shared in this article will need to be determined over time with site specific process evaluation and outcome metrics. However, the models presented here provide epilepsy center teams with guidance for action as currently the known skills and resource acumen which a CHW workforce could provide to patients and families is severely underutilized in epilepsy care settings.

We recognize the limitation that the CHW integration model presented here is based on epilepsy center environments in the U.S. health system and do not address applicability in international epilepsy care settings. This project was a Centers for Disease Control and Prevention supported effort with a domestic focus per Congressional program directives. Applicability of these models in international settings and contexts will require additional efforts that consider variables such as universal health systems and challenges (e.g., training systems, regulatory systems, funding mechanisms, existing roles structure and the potential for integration) which may present in low and middle income countries (LMIC). Despite these challenges there is good evidence of the integration of non-medical providers inclusive of CHWs in chronic disease care streams in LMIC; and interventions that contextually advance training and implementation of CHW services are growing in regard as effective approaches to address capacity constraints faced in low resource settings, including in epilepsy, are explored (38–44).

## 5 Conclusion

The pivotal role of CHWs in improving patient outcomes, quality of care, and cost-effectiveness has been demonstrated in over a decade of research. Evaluation of our models on a wider scale in unique epilepsy center settings will be necessary to fully evaluate effectiveness, costs, and implementation in different contexts.

## Data Availability

The original contributions presented in the study are included in the article/supplementary material, further inquiries can be directed to the corresponding author.

## Footnotes

^1^See https://nachw.org/generalresources/networks-and-training-programs/

^2^See https://managingepilepsywell.org/community-health-worker

^3^See https://www.dartmouth-hitchcock.org/hobscotch-institute/community-training

^4^See https://nashp.org/state-tracker/state-community-health-worker-policies/kentucky/; https://nashp.org/state-tracker/state-community-health-worker-policies/georgia/; https://www.cthealth.org/publication/50-state-scan-of-medicaid-payment-for-community-health-workers/
